# Supplementary material for: Resolving the full spectrum of human genome variation using Linked-Reads
Source: Genome Res. 2019 Apr;29(4):635–45. doi: 10.1101/gr.234443.118 (PMC6442396; doi:10.1101/gr.234443.118)
Supplement: Supplemental Material [file supp_gr.234443.118_Supplemental_longranger-2.2.zip › longranger-2.2.tar.bz2!/lr2.2-public/lib/stan/cmdstan/stan/doc/api/html/classstan_1_1mcmc_1_1ps__point-members.html]

Stan: Member List


|  |  |
| --- | --- |
|  | Stan  2.5.0  probability, sampling & optimization |


- Main Page
- Related Pages
- Namespaces
- Classes
- Files

- Class List
- Class Index
- Class Hierarchy
- Class Members

All Classes Namespaces Files Functions Variables Typedefs Enumerations Enumerator Friends Macros Pages

- stan
- mcmc
- ps\_point

stan::mcmc::ps\_point Member List

This is the complete list of members for stan::mcmc::ps\_point, including all inherited members.

|  |  |  |
| --- | --- | --- |
| fast\_matrix\_copy\_(Eigen::Matrix< T, Eigen::Dynamic, Eigen::Dynamic > &v\_to, const Eigen::Matrix< T, Eigen::Dynamic, Eigen::Dynamic > &v\_from) | stan::mcmc::ps\_point | inlineprotectedstatic |
| fast\_vector\_copy\_(Eigen::Matrix< T, Eigen::Dynamic, 1 > &v\_to, const Eigen::Matrix< T, Eigen::Dynamic, 1 > &v\_from) | stan::mcmc::ps\_point | inlineprotectedstatic |
| g | stan::mcmc::ps\_point |  |
| get\_param\_names(std::vector< std::string > &model\_names, std::vector< std::string > &names) | stan::mcmc::ps\_point | inlinevirtual |
| get\_params(std::vector< double > &values) | stan::mcmc::ps\_point | inlinevirtual |
| operator=(const ps\_point &z) | stan::mcmc::ps\_point | inline |
| p | stan::mcmc::ps\_point |  |
| ps\_point(int n) | stan::mcmc::ps\_point | inline |
| ps\_point(const ps\_point &z) | stan::mcmc::ps\_point | inline |
| ps\_point\_test class | stan::mcmc::ps\_point | friend |
| q | stan::mcmc::ps\_point |  |
| V | stan::mcmc::ps\_point |  |
| write\_metric(std::ostream \*o) | stan::mcmc::ps\_point | inlinevirtual |


---

[ Stan Home Page ]

*© 2011–2014,
Stan Development Team.*
